# Influence of preoperative life satisfaction on recovery and outcomes after colorectal cancer surgery - a prospective pilot study

**DOI:** 10.1186/s12955-017-0824-4

**Published:** 2018-01-17

**Authors:** B. Romain, O. Rohmer, S. Schimchowitsch, M. Hübner, J. B. Delhorme, C. Brigand, S. Rohr, D. Guenot

**Affiliations:** 1 Université de Strasbourg, EA 3430, Tumor progression and microenvironment. Translational approaches and epidemiology, 3 avenue Molière, 67200 Strasbourg, France; 2 Department of Digestive Surgery, Strasbourg University Hospital, 1 Avenue Moliere, 67000 Strasbourg, France; 3 Laboratoire de Psychologie des Cognitions, Strasbourg University, Strasbourg, France; 4 Department of Visceral Surgery, University Hospital CHU, 1011 Lausanne, Switzerland

**Keywords:** Life satisfaction, Colorectal cancer surgery, Rehabilitation, Postoperative complications, Pain, Sleep

## Abstract

**Background:**

Colorectal surgery has an important impact on a patient’s quality of life, and postoperative rehabilitation shows large variations. To enhance the understanding of recovery after colorectal cancer, health-related quality of life has become a standard outcome measurement for clinical care and research. Therefore, we aimed to correlate the influence of preoperative global life satisfaction on subjective feelings of well-being with clinical outcomes after colorectal surgery.

**Methods:**

In this pilot study of consecutive colorectal surgery patients, various dimensions of feelings of preoperative life satisfaction were assessed using a self-rated scale, which was validated in French. Both objective (length of stay and complications) and subjective (pain, subjective well-being and quality of sleep) indicators of recovery were evaluated daily during each patient’s hospital stay.

**Results:**

A total of 112 patients were included. The results showed a negative relationship between life satisfaction and postoperative complications and a significant negative correlation with the length of stay. Moreover, a significant positive correlation between life satisfaction and the combined subjective indicators of recovery was observed.

**Conclusion:**

We have shown the importance of positive preoperative mental states and global life satisfaction as characteristics that are associated with an improved recovery after colorectal surgery. Therefore, patients with a good level of life satisfaction may be better able to face the consequences of colorectal surgery, which is a relevant parameter in supportive cancer care.

## Background

Surgery, particularly colorectal cancer surgery, has an important impact on a patient’s quality of life [1], and postoperative rehabilitation can be difficult to handle [2, 3]. The classic medical outcomes for surgical procedures are usually mortality, complications, recurrence rate and long-term survival. However, from the patient’s perspective, the length of the hospital stay, convalescence duration, subjective well-being, sensation of pain, and quality of sleep are just as important [4–6]. In an attempt to improve patient management and health recovery, standardized multimodal perioperative care programmes were recently introduced, particularly in the framework of colorectal cancer [7]. These protocols, involving both the participants in the care and the patients themselves, were designed to achieve enhanced recovery after surgical (ERAS) procedures by maintaining organ function and reducing a profound stress response following surgery [7, 8]. ERAS protocols place the patient’s recovery in the centre of perioperative care and have led to considerable improvements in easily measurable outcomes such as complications (−40%) and hospital stays (−2.5 days) [8, 9].

However, little has been reported on actual recovery. The reason for this is that recovery is poorly defined and that the available scores can present with certain limitations, especially because a consensus is lacking on the available criteria [10].

To enhance the understanding of recovery after colorectal cancer, health-related quality of life has become a standard outcome measurement in clinical care and research. Attention to this parameter from both patients and surgeons has already led to surgical innovations that aim to preserve sphincters, mitigate faecal disorders and reduce the risk for sexual dysfunction [11]. However, an appropriate evaluation of health-related quality of life after surgery should take a wider range of markers into account [12] from both objective (i.e., physical activity) and subjective (i.e., psychological feelings) standpoints. Quality of life after surgery has been studied using different scales; after colorectal cancer, for example, the EORTC QLQ-C30 has been recommended to measure postoperative recovery [13]. However, in this type of questionnaire, concepts linked to environmental factors, one’s place in society, relationships, and, more broadly, general feelings of life satisfaction are neglected. Nevertheless, recent reports have described the relationship between psychological and emotional characteristics prior to a stressful event, such as surgery, and the ways in which individuals respond during the recovery period [14–16]. Moreover, life satisfaction, which refers to human characteristics that are stable over time (and thus not to transient states), was shown to significantly impact the recovery process in a heterogeneous sample of patients [17].

Thus, in terms of clinical applications, the extent to which a patient’s satisfactory assessment of his/her well-being and quality of life before surgery will play a role in alleviating some postoperative outcomes appears to be important for further investigations. Notably, progress in this area may foster the optimization of ERAS protocols to better take into account a patient’s psychological care. Therefore, the aim of this pilot study, which focused on colorectal cancer patients, was to analyse the influence of subjective feelings on various areas of life on different objective and subjective outcomes after colorectal surgery, which was assessed as a global component of life satisfaction [18].

## Methods

### Procedure

This prospective pilot study included a consecutive cohort of patients undergoing elective colorectal cancer surgery, regardless of clinical stage or relapse event, from March 2015 to December 2015 at the Visceral Surgical Department of Hautepierre University Hospital (France). All the patients (*n* = 112) were informed about the study at their first consultation, and they all agreed to participate and signed a consent form. They were treated according to the ERAS (*Enhanced Recovery After Surgery)* protocol [7]. A dedicated and specially trained enhanced recovery nurse was in charge of completing the prospective database (*ERAS Interactive Audit System)* and of collecting the demographic and surgical details of all the patients in the enhanced recovery pathway, recording daily detailed information of clinical outcomes until a maximum of 30 days after surgery.

The institutional review board approved the study, and all the patients provided written consent before the surgery. Whenever possible, the STROBE criteria that are compatible with the small sample size of this pilot study were applied.

### Measures

During the first consultation, preoperative life satisfaction was assessed with the Canadian *Échelle de Mesure des Manifestations du Bien-Être Psychologique* (EMMBEP) scale [18], which is a validated tool with a high internal consistency (alpha = 0.93) [18] that has already been applied to the French population [19] in the assessment of positive feelings in the following six various areas of life: attitudes towards the self (e.g., “I feel useful”), relations with others - close and open relationships (e.g., “I feel loved and appreciated”), self-regulation (e.g., “I feel balanced”), environmental mastery (e.g., “I face difficulties with positivity”), sociability (e.g., “I keep my friends laughing”) and happiness (e.g., “I feel good”). The respondents scored each of the 25 items using a 5-point scale varying from 0 (“strongly disagree”) to 4 (“strongly agree”). The internal consistency of this scale in the study population was calculated by the Cronbach’s alpha coefficient.

The preoperative physical activity of each patient was assessed according to the WHO’s recommendations using the self-reported Saltin-Grimby Physical Activity Level Scale [20]. However, due to the very small number of patients potentially concerned with competition sports, the categories “Regular physical activity and training” and “Regular hard physical training for competition sports” were merged into one [20]. Thus, the three remaining answer categories were scored from 0 (physically inactive: sedentary activities during leisure-time such as reading, watching television or movies, using computer, etc.) to 2 (intensive regular physical activity: running, swimming, playing tennis, badminton, or similar activities for at least 2 to 3 h/week).

### Recovery factors

#### Objective factors

Postoperative complications were graded according to the Dindo-Clavien classification system [21], which enables a ranking of the complications in an objective and repeatable manner; medical complications were classified as grade I (least severe complication) or II, whereas complications requiring surgical treatment were classified as grade III. Patients with life-threatening complications or patients who died were graded as IV and V, respectively. The length of stay was counted from the day of surgery until discharge.

#### Subjective factors

Subjective indicators of recovery were evaluated daily by the nurse involved in the enhanced recovery program during the hospital stay; pain, quality of sleep and well-being were assessed through the use of easily deliverable visual analogue scales (VAS) from 0 (no pain, poor sleep, poor postoperative well-being) to 10 (major pain, good sleep, good postoperative well-being). The pain scores were reversed (no pain = 10, major pain = 0) to match the two other indicators so that the high scores could reflect enhanced recovery. These three subjective scores were combined into one subjective recovery indicator.

As the minimal length of stay after the surgery was 4 days for more than 10% of the patients, we only retained the scores recorded during the first 4 days post-surgery for a coherent sample analysis. These scores were averaged for each subjective indicator.

### Statistical analysis

The data analyses were performed using Statistica 12 (DELL Software International), and a conventional level of 0.05 was used to indicate statistical significance. As shown in Table 1, the descriptive statistics for the categorical variables are reported as frequencies (%), while the continuous variables are reported as the means (standard deviation, SD). To analyse the statistical group comparisons with F (Analysis of Variance) statistic, the normal distribution of the indicators was tested with the Kolmogorov-Smirnov test. Bivariate correlations between these continuous indicators were assessed using the r statistic (Pearson Test), whereas relations between the discrete indicators were tested with the chi^2^ statistic.

**Table 1:** Descriptive statistics of the outcomes assessed before and after the surgery (without any standardization or transformation)

Preoperative life satisfaction [1–4]	
Mean score ± SD	3.02 ± 0.58
Preoperative physical activity (0,1,2)	
Frequency 0	3.61%
Frequency 1	39.16%
Frequency 2	56.70%
Complications	
Frequency	50%
- Grade 1	14.3%
- Grade 2	21.4%
- Grade 3	11.6%
- Grade 4	2.7%
- Grade 5	0
Length of stay [4–37]	
Mean score ± SD	10.0 ± 7.84
Subjective pain [0–10]	
Mean score ± SD	3.44 ± 1.69
Subjective sleep [0–10]	
Mean score ± SD	4.59 ± 1.72
Subjective well-being [0–10]	
Mean score ± SD	5.78 ± 1.39

An exploratory principal component analysis of the correlation matrix of the objective and subjective recovery indicators (eigenvalue >1) was performed to determine the subscales that could be used as variables for inferential statistics. This analysis is recommended to be performed at the preliminary step of research when there is no hypothesis about the relevant dimensions prior to the data collection. Principal component analysis produces a small set of uncorrelated components based on the scores of different measures to provide an operational definition for an underlying process. The components that are highlighted by this analysis empirically summarize the correlations among the different measures. This type of analysis allows the researcher to determine the coordinates of each participant for each factor extracted, and then these factorial scores can be used as dependent variables for inferential statistics. Such empirical composite scores are estimated to be more reliable than scores on individually observed variables, especially when subjective measures are involved. To permit this preliminary analysis on different measures of recovery using very different units, z-scores (normal random variables of standard distributions) were calculated for each measure.

For the analyses, life satisfaction was correlated with recovery factors. However, in a previous step, by using the median score as the cut-off criterion we stratified the population into a low-level life satisfaction group and a high-level one. This stratification enabled us to ensure that both groups did not differ in terms of gender, age, intervention or approach.

## Results

The final analysis included 112 consecutive colorectal patients. The participants (55% males) were aged from 18 to 90 years (*M*_*age*_ = 63.79 years, *SD*_*age*_ = 13.93).

First, it is important to note that the low-level life satisfaction group and the high-level one (median score: 3.09) can be considered as equivalent in regard to their individual characteristics; the results showed no difference in terms of age (*p* = 0.64), gender (*p* = 0.80), intervention (*p* = 0.45), or approach (Laparoscopy: *p* = .45; Open: *p* = 0.37; Conversion: *p* = 0.34). Not surprisingly, the results indicated that the patients with the high preoperative physical activity had a significantly higher life satisfaction score than those with the lower physical activity (*p* = 0.02) (see Table 2).

**Table 2:** Comparisons between low life satisfaction patients and high life satisfaction patients (using the median score as the cut-off criterion) in terms of age, gender, intervention, approach and physical activity

Individual characteristics	Low life satisfaction [1125–3094]	High life satisfaction [3095–4]	*p* value
Age (*M*_years_ ± *SD*)	64.6 ± 14.4	62.9 ± 13.5	0.64
Gender (M/F)	31/25	30/26	0.80
Intervention (*N*)			
- Right colectomy	8	11	0.45
- Left colectomy	12	10	
- Rectal resection	9	5	
- Abdominoperineal resection	3	5	
- Small bowel resection	2	1	
- Other	13	15	
- Stoma closure	4	8	
- HIPEC	5	1	
Approach (*N*)			
- Laparoscopy	19	23	0.45
- Open	35	30	0.37
- Conversion	5	4	0.34
Preop. physical activity (*N*)			
- None	28	16	0.02
- Moderate	25	38	
- Intensive	1	2	
- Unknown	2	0	

### Life satisfaction

After verifying that no relationship between life satisfaction and the individual variables existed, we used the raw scores to conduct correlational analyses between life satisfaction and the different indicators of recovery. As the internal consistency of the EMMBEP scale was high (*Cronbach’s alpha coefficient* = 0.86), all the items of this scale were combined into a single measure of life satisfaction. Overall, the preoperative life satisfaction was 3.02 (*SD* = 0.58), with a normal distribution of the data ranging from 0 to 5 (Normality: *p* = 0.20). Furthermore, 55.3% of the patients obtained a score greater than the mean.

### Indicators of recovery

The Bartlett’s test of sphericity was applied to the correlation matrix, showing any multicollinearity (*p* = 0.0001). Thus, a principal factor extraction with Varimax rotation was conducted, and two factors were extracted that accounted for 56.19% of the total variance. The first factor comprised the following three subjective measures: pain (*loading*: 0.40), sleep (−0.86), and well-being (−0.66). The second factor comprised the following two objective indicators: length of stay (0.88) and complications (0.90).

### Relations between life satisfaction and subjective and objective recovery indicators

**Fig. 1:**
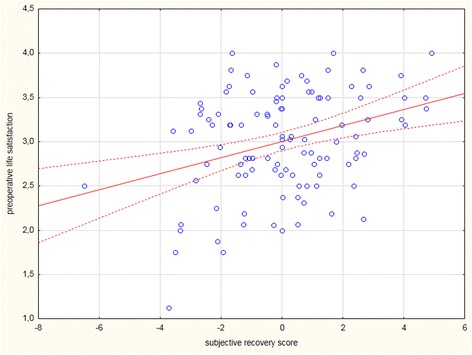
Correlation between preoperative life satisfaction scores and postoperative subjective recovery indicator. The latter combines the sleep, pain and well-being scores *(r* = 0.33*; p* = 0.0001*).* Dotted lines display confidence limits (confidence interval of 95%)

Life satisfaction was significantly correlated with the subjective recovery scores (*p* = 0.0001) (Fig. 1). The patients with a higher life satisfaction before surgery felt better. Indeed, overall, these patients reported a combination of less pain, better sleep or better feelings. Nevertheless, life satisfaction was not significantly correlated with the objective recovery scores (*p* = 0.19). We further examined each clinical outcome separately.

Overall, the complication rate according to the Clavien classification was 50% (*N* = 56). The levels of the complications (Grades 1 to 5) are highlighted in Table 1. Patients had fewer complications when they expressed a higher preoperative life satisfaction, but this relation failed to reach the conventional level of significance (*p* = 0.06).

**Fig. 2:**
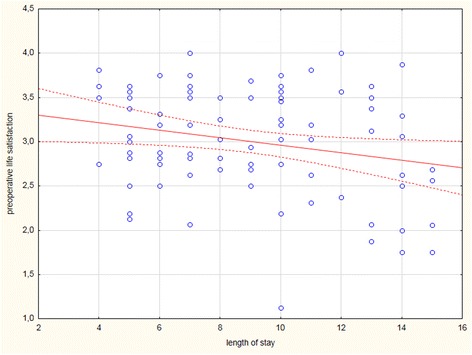
Correlation between preoperative life satisfaction scores and the length of stay (days) *(r =* 0.23*; p* = 0.03*)*. Dotted lines display confidence limits (confidence interval of 95%)

Overall, the median length of stay was 10 days (from a minimum of 4 days to a maximum of 37 days); only 17% of the participants were hospitalized for more than 15 days. There was a significant correlation between preoperative life satisfaction and the length of stay during the first 15 postoperative days (*p* = 0.03). The higher the preoperative life satisfaction was, the lower the length of stay (Fig. 2).

## Discussion

Recent literature has noted the importance of quality of life as a relevant factor of recovery after surgery. In a number of studies, health-related quality of life was mostly restricted to physical and medical outcomes after surgical procedures, such as mortality or complications [22–27]. However, the influence of different personality or subjective feeling factors related to psychological well-being has recently been singled out [15–17, 28]. Focusing on a sample of colorectal cancer patients, the aim of this pilot study was to evaluate how preoperative feelings of global life satisfaction can significantly influence recovery outcomes after surgery. To properly assess these outcomes, a set of both objective and subjective markers of recovery were taken into account [12]. We have shown that positive preoperative life satisfaction was a significant factor that was correlated with a better postoperative rehabilitation, namely, it was associated with a significantly decreased length of stay combined with a trend of reduced postoperative complications. Furthermore, preoperative life satisfaction was significantly related with the postoperative subjective marker of recovery, which combines well-being, quality of sleep and pain sensation. It also seems that preoperative life satisfaction appears to be a particularly good predictor of subjective well-being after surgery; at this point in our research, the positive effects more strongly appeared to be subjective indicators of recovery rather than objective ones.

Our results are in accordance with previous works showing that negative feelings and emotional distress constitute major barriers to recovery from cancer [29]. In the context of health schemes and of efforts to reduce morbidity and mortality, special attention is currently being paid to understand and improve the preoperative factors affecting postoperative recovery. As coping behaviour [17] and patient activation, i.e., one’s propensity to engage in positive health, have been identified as important modifiers of the recovery process [28], proactive approaches have now been put forward. In this same vein, some attempts at preoperative strategies that are based on cognitive-behavioural patient education, acceptance-based nursing intervention, exercise training or pre-habilitation have been initiated and described to help optimize postoperative recovery [30–34]. Such research notes the importance of patients being provided with early psychological support and being helped to develop self-management skills to better cope with illness and surgical outcomes [35]. Insofar as preoperative life satisfaction may represent a key point in the fostering of health-related recovery, it would be interesting to implement appropriate interventions that are able to improve this parameter. This would provide a valuable resource to patients, especially those with negative mood and emotional distress, both of which present a greater post-surgical risk for continued physical disability [16]. In this context, mindfulness-based interventions (MBI) could be particularly effective [36]. Indeed, a growing number of studies concerning coping with cancer, have reported a broad spectrum of the positive effects of MBI, ranging from quality of life domains (emotional, social and physical functioning) to psychological improvements (reduced symptoms of depression, anxiety, insomnia…) and biological markers of health [11]. However, the MBI in these studies were mostly concerned with ongoing care patients, as well as with survivors of cancer. It would be interesting to test the effects of earlier mindfulness training, particularly on the improvement of the parameters of the feelings of life satisfaction in preoperative patients with colorectal cancer.

Our pilot study presented several limitations due to a modest and heterogeneous patient cohort and to the absence of a life satisfaction estimation in a safe patient cohort. In this exploratory research, we were not able to reflect the sublevels of the pathologies of the interventions. Moreover, our correlational perspective did not allow us to conclude cause-effect relationships. Furthermore, the subjective tools and scores deserve to be complemented by more objective measures and conventional scales. However, this is the first study, to our knowledge, in the field of colorectal cancer that correlated preoperative feelings of life satisfaction with objective and subjective postoperative factors.

## Conclusion

We have highlighted the importance of positive preoperative subjective states and global feelings of life satisfaction as characteristics that are associated with improved subjective, as well as clinical, outcomes after colorectal surgery. Such parameters deserve to be taken into account to optimize multimodal ERAS care protocols that have been recently introduced to foster recovery. Notably, the strengthening of well-being and life satisfaction by early interventions, such as MBI, could be effective aides for patients to cope and thrive through the difficult challenge of colorectal cancer. The strategy to improve a patient’s chance of a successful outcome by providing more comprehensive perioperative care should be especially valuable in the context of health schemes. This area of research and clinical implications deserves to be further explored.

## Abbreviations

CCR: colorectal cancer
EMMBEP: Échelle de Mesure des Manifestations du Bien-Être Psychologique
ERAS: Enhanced Recovery After Surgery
LS: Life satisfaction
MBI: Mindfulness based intervention
VAS: visual analogue scales
